# Physical activity during pregnancy stratified by body mass index: A focus on class 3 obesity

**DOI:** 10.1002/pmf2.70083

**Published:** 2025-07-30

**Authors:** Eliza M. Nguyen, Megan E. Branda, Linda M. Szymanski

**Affiliations:** ^1^ Department of Obstetrics & Gynecology Mayo Clinic Rochester Minnesota USA; ^2^ Mayo Clinic Rochester Minnesota USA

**Keywords:** body mass index, class 3 obesity, exercise, morbid obesity, obesity, physical activity, severe obesity

## Abstract

**Introduction:**

The prevalence of pre‐pregnancy obesity, including pre‐pregnancy class 3 obesity, has increased over time, with significant implications for maternal and neonatal well‐being. Regular physical activity may mitigate these risks. Available data suggest that most pregnant individuals do not meet the recommended physical activity guidelines of accumulating at least 150 min of moderate‐intensity exercise per week. Importantly, limited data are available regarding physical activity patterns in individuals with higher body mass index (BMI), particularly for those with class 3 obesity (BMI ≥ 40 kg/m^2^). The aim of this study was to evaluate whether pregnant individuals are meeting physical activity guidelines. We assessed self‐reported physical activity across BMI categories including those with class 3 obesity, further subdivided into BMI of 40–49.9 and ≥ 50 kg/m ^2^.

**Methods:**

This is a retrospective cohort study of individuals at an academic hospital system who delivered between May 1, 2018 and May 1, 2023. To be included, individuals had to respond to the following two‐item physical activity questionnaire early in pregnancy: (1) “On average, how many days per week do you engage in moderate to strenuous exercise?” And (2) “On average, how many minutes do you engage in exercise at this level?” From these two questions, total minutes per week of activity were calculated. Individuals met physical activity guidelines if they engaged in ≥ 150 min of physical activity per week. Data were analyzed using Kruskal–Wallis and Chi‐square tests.

**Results:**

A total of 6884 individuals were incl uded in the cohort. A total of 510 individuals met the criteria for class 3 obesity, with 61 having a pre‐pregnancy BMI ≥ 50 kg/m ^2^. Among normal weight individuals (BMI 18.5–24.9 kg/m ^2^), 34% met physical activity guidelines. Overweight and obese individuals were significantly less physically active than those with normal BMI (*p* < 0.05). Only 22% of those with BMI 40–49.9 kg/m ^2^ and 18% of those with BMI ≥ 50 kg/m ^2^ reported sufficient physical activity.

**Conclusion:**

Our results are consistent with existing data, showing that pregnant individuals are not meeting physical activity goals. Pregnant individuals who are overweight or obese engage in significantly less physical activity compared to those with a BMI in the normal range, with the lowest levels of activity observed among individuals with class 3 obesity. More data are needed on physical activity among pregnant individuals with class 3 obesity, including evaluation of potential interventions to increase activity as well as barriers to participation. Increasing physical activity in this group may positively impact both maternal and neonatal health.

## 1 INTRODUCTION

Rates of obesity in the United States have been increasing over the past 40 years [1, 2]. Between 1988 and 2018, the overall prevalence of obesity, defined as a body mass index (BMI) ≥ 30 kg/m^2^, increased from 22.9% to 42.4%. The prevalence of class 3 obesity, defined as BMI ≥ 40 kg/m^2^, more than tripled during this period, rising from 2.8% to 9.2%, with females accounting for a greater proportion of this increase than males [2]. Between 2021 and 2023, the prevalence of women aged 20–39 with class 3 obesity was 13% [3].

Pre‐pregnancy obesity has followed a similar trend [4]. According to data from the National Vital Statistics, the rate of pre‐pregnancy obesity increased from 25.4% to 30.9% between 2016 and 2022; those with pre‐pregnancy BMI ≥ 40 kg/m^2^ increased from 4.8% to 6.1% [5]. This is concerning because obesity and excessive gestational weight gain have been associated with adverse maternal and neonatal outcomes, particularly for those on the upper extreme of BMI. Associated adverse maternal outcomes include increased risks of miscarriage, gestational diabetes, hypertensive disorders of pregnancy, prolonged labor course, infection, cesarean delivery, unplanned hysterectomy, venous thrombosis, and ICU admission. Neonatal complications include an increased risk of congenital anomalies, being either too large or small for gestational age, preterm birth, stillbirth, need for respiratory support, and ICU admission. Additionally, affected offspring are at increased risk for childhood obesity and metabolic disorders [6, 7, 8, 9].

Engaging in regular physical activity during pregnancy has been associated with numerous maternal health benefits, including decreased rates of gestational diabetes mellitus, excessive weight gain, hypertensive disorders, cesarean and operative vaginal birth, peripartum mood disorders, and postpartum weight retention [10, 11, 12, 13, 14, 15, 16]. Additionally, prenatal exercise has been shown to confer fetal and neonatal benefits, such as decreased risk of macrosomia (birthweight over 4000 g), without raising the likelihood of preterm birth or low birthweight [17]. The *Physical Activity Guidelines for Americans* issued by the US Department of Health and Human Services recommend at least 150 minutes of moderate intensity aerobic exercise per week during both pregnancy and the postpartum period [18]. This recommendation is also supported by the American College of Obstetricians and Gynecologists (ACOG) and the Society of Obstetricians and Gynaecologists of Canada (SOGC); however, adherence to this guideline is low [10, 19]. Studies in the United States demonstrate that only 3%–15% of pregnant individuals meet these physical activity guidelines, with even lower rates observed in those with obesity compared to those without [20, 21, 22, 23]. Furthermore, data specific to pregnant individuals with class 3 obesity are limited as existing studies frequently aggregate all participants with a BMI ≥ 30 kg/m^2^ into a single category, thereby limiting subgroup analyses and potentially overlooking important distinctions among those with higher BMI [24, 25, 26].

The aims of this study were twofold: (1) to ascertain the percentage of pregnant individuals who are meeting physical activity guidelines and (2) to determine if there are differences in physical activity when stratified by BMI. In this study, we go beyond the traditional BMI classifications to include data specifically on individuals with BMI 40–49.9 and ≥ 50 kg/m^2^ to more closely evaluate this understudied population.

## 2 MATERIALS AND METHODS

This was a retrospective cohort study at an academic hospital and its affiliated satellite sites, which share an electronic medical record system. Patients that delivered between May 1, 2018 and May 1, 2023 were included. Inclusion criteria included age ≥18 years, singleton pregnancy, and completion of a self‐reported measure of physical activity between the time of conception until 20 weeks gestational age. Exclusion criteria include missing pre‐gravid BMI and pregnancies that did not progress beyond 20 weeks gestational age. Research authorization must be provided by participants to be included in the analysis. This study was determined to be exempt from the requirement for IRB approval (IRB # 22‐008070).

### 2.1 Data

At our institution, patients are routinely asked to complete intake surveys as part of their medical care. These surveys include patient demographics, medical history, and social determinants of health, which include two questions on physical activity: (1) “On average, how many days per week do you engage in moderate to strenuous exercise (like a brisk walk)?” and (2) “On average, how many minutes do you engage in exercise at this level?” Potential responses to these questions were the following: (1) 0–7 days in single day increments and (2) 0–150+ minutes in 10‐minute increments (i.e., 0 min, 10 min, 20 min… 150+ min). Responses to these two items were then multiplied to provide the number of minutes of physical activity per week. Physical activity was classified as either ≥150 min per week or < 150 min per week. Survey items were collected between the date of conception and 20 weeks gestational age. If survey items were collected multiple times during that time period, the earliest response was used. Pre‐gravid BMI was determined using the weight recorded closest to date of conception, within a window of 60 days before or after. Delivery BMI was based on weight obtained up to 14 days prior to delivery.

### 2.2 Outcomes

The primary outcome of interest was the percentage of individuals in each BMI category who met the recommended physical activity guidelines. After calculating minutes of physical activity per week for each BMI category, participants were then classified as either meeting guidelines if they achieved ≥150 min of exercise per week or not meeting guidelines. We used the following BMI categories: underweight (< 18.5 kg/m^2^), normal (18.5–24.9 kg/m^2^), overweight (24.9–29.9 kg/m^2^), obese class 1 (30–34.9 kg/m^2^), obese class 2 (35–39.9 kg/m^2^), obese class 3 (40–49.9 kg/m^2^), and obese class 3 (≥50 kg/m^2^).

### 2.3 Statistical analyses

Descriptive statistics include means and standard deviations for continuous variables and counts and frequencies for categorical variables. Comparisons between groups were conducted using the Kruskal–Wallis test for continuous variables and the Chi‐square test for categorical variables.

## 3 RESULTS

A total of 6884 individuals were included in the cohort. A total of 510 individuals met criteria for class 3 obesity: 449 had a BMI 40–49.9 kg/m^2^ and 61 had a BMI ≥ 50 kg/m^2^. Demographic data are presented in Table 1. Patients were primarily non‐Hispanic, White, English‐speaking, and multiparous. There were no group differences in maternal age. There was a significant difference in gestational weight gain between the groups: higher BMI was associated with lower gestational weight gain. Gestational age at delivery and neonatal birthweight were also significantly different between groups, with early delivery and higher rates of large for gestational age neonates for individuals in higher BMI categories.

**TABLE 1:** Demographics by BMI category.

	Participant Characteristics by BMI Category							
	Underweight (*N* = 122)	Healthy (*N* = 2604)	Overweight (*N* = 1876)	Obese Class 1 (*N* = 1117)	Obese Class 2 (*N* = 655)	Obese Class 3: 40 to < 50 (*N* = 449)	Obese Class 3: 50+ (N = 61)	*p* value
BMI at conception Mean (SD)	17.7 (0.74)	22.2 (1.67)	27.3 (1.44)	32.3 (1.46)	37.3 (1.44)	43.4 (2.60)	54.4 (4.91)	<.01^a^
Maternal age at delivery Mean (SD)	29.4 (4.80)	30.3 (4.87)	30.1 (4.93)	30.1 (4.94)	30.3 (4.99)	30.0 (5.15)	30.5 (4.30)	0.15^a^
Gestational age at delivery (w) Mean (SD)	38.5 (2.69)	38.6 (1.97)	38.5 (2.20)	38.4 (2.40)	38.3 (2.64)	38.0 (2.57)	37.8 (1.78)	<.01^a^
Gestational weight change (kg)	13.2 (6.41)	13.0 (6.93)	12.1 (7.56)	9.7 (8.28)	7.8 (8.53)	7.2 (8.36)	6.7 (8.99)	<.01^a^
Birthweight Category *n* (%)								<.01^a^
Small for gestational age	20 (18%)	193 (8%)	108 (6%)	56 (5%)	21 (3%)	20 (5%)	5 (8%)	
Appropriate for gestational age	85 (77%)	1927 (81%)	1388 (80%)	794 (77%)	451 (74%)	313 (73%)	36 (61%)	
Large for gestational age	3 (3%)	255 (11%)	236 (14%)	181 (18%)	136 (22%)	92 (22%)	18 (30%)	
Gestational age < 24 weeks	2 (2%)	5 (0%)	6 (0%)	2 (0%)	1 (0%)	0 (0%)	0 (0%)	
Race, *n* (%)								<.01^b^
White	102 (84%)	2283 (89%)	1648 (89%)	1003 (90%)	586 (91%)	410 (92%)	56 (93%)	
Black	7 (6%)	75 (3%)	80 (4%)	49 (4%)	33 (5%)	23 (5%)	3 (5%)	
Asian	13 (11%)	156 (6%)	89 (5%)	22 (2%)	8 (1%)	6 (1%)	1 (2%)	
AI/AN	0 (0%)	6 (0%)	7 (0%)	13 (1%)	5 (1%)	4 (1%)	0 (0%)	
NH/PI	0 (0%)	7 (0%)	2 (0%)	3 (0%)	2 (0%)	1 (0%)	0 (0%)	
Other	0 (0%)	50 (2%)	29 (2%)	21 (2%)	12 (2%)	2 (0%)	0 (0%)	
Highest level of school attended *n* (%)								<.01^b^
Less than Highschool graduate	25 (22%)	215 (9%)	188 (11%)	155 (15%)	102 (16%)	82 (19%)	16 (28%)	
Highschool Graduate/GED	10 (9%)	150 (6%)	127 (7%)	91 (9%)	55 (9%)	58 (13%)	5 (9%)	
Associates/Some college	25 (22%)	533 (22%)	541 (31%)	351 (34%)	248 (40%)	164 (38%)	23 (40%)	
Bachelors	31 (28%)	916 (38%)	598 (34%)	318 (30%)	158 (25%)	98 (23%)	12 (21%)	
Graduate/Professional degree	21 (19%)	582 (24%)	294 (17%)	131 (13%)	62 (10%)	30 (7%)	2 (3%)	
Pre‐existing Diabetes, *n* (%)	0 (0%)	21 (1%)	31 (2%)	36 (3%)	28 (4%)	35 (8%)	7 (11%)	<.01^b^
Chronic Hypertension, *n* (%)	1 (1%)	46 (2%)	55 (3%)	76 (7%)	67 (10%)	82 (18%)	20 (33%)	<.01^b^
Polycystic ovary syndrome, *n* (%)	2 (2%)	50 (2%)	45 (2%)	49 (4%)	47 (7%)	33 (7%)	5 (8%)	<.01^b^

^a^ Kruskal–Wallis *p* value.

^b^ Chi‐square *p* value.

**FIGURE 1:**
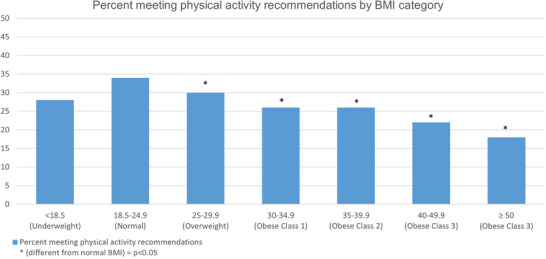
Percent of individuals meeting recommended physical activity guidelines by BMI category.

Among those with a normal BMI, 34% met the recommendation of at least 150 min of weekly physical activity. Except for those classified as underweight, all other BMI categories reported less physical activity, with significantly fewer individuals reaching the recommended threshold. Specifically, 22% of individuals with BMI 40–49.9 kg/m^2^ and 18% of those with a BMI of 50 kg/m^2^ or higher met the guideline (Figure 1). Those with a normal BMI exercised an average of 126.5 minutes per week. For individuals with class 3 obesity, average minutes of activity per week were 101.2 and 107.7 for BMI 40–49.9 kg/m^2^ and BMI ≥ 50 kg/m^2^, respectively.

## 4 DISCUSSION

In this retrospective analysis of physical activity during pregnancy, the majority of pregnant individuals did not meet the recommended physical activity guidelines. An inverse relationship was observed between physical activity and BMI, with rates of adequate physical activity decreasing as BMI increased. Those with class 3 obesity exercised less and were less likely to achieve the recommended amount of weekly physical activity compared to their non‐obese counterparts, and those with a BMI of 50 kg/m^2^ and higher met the weekly activity recommendations at the lowest rates within the cohort.

Our data are consistent with existing literature demonstrating that pregnant patients are not achieving the recommended amount of physical activity [20, 21, 22, 23]. Further, our findings also align with prior studies showing that pregnant individuals with obesity tend to be even less active than their normal weight counterparts [24, 25, 26]. A novel component of our study is that we stratified participants by BMI category, allowing for a more detailed analysis compared to prior studies that commonly group all individuals with obesity into a single group encompassing classes 1, 2, and 3. Thus, there is very limited data addressing individuals with a BMI ≥ 40 kg/m^2^ and data on those with a BMI ≥ 50 kg/m^2^ is essentially nonexistent.

For example, Amezcua‐Prieto et al. demonstrated that among a cohort of pregnant individuals in Spain between 20 and 22 weeks gestational age, 12.7% of those with obesity satisfied the recommended physical activity guideline of at least 30 min of moderate exercise on 5 days per week, compared to 21.7% of those with a normal BMI [24]. However, the study included 118 individuals with obesity, defined as a BMI ≥ 30 kg/m^2^ without further stratification. Similarly, Andersen et al. evaluated step counts and metabolic equivalent of tasks (METs) in a Danish cohort of 400 pregnant individuals [25]. Consistent with our findings, they demonstrated an inverse linear relationship between physical activity and BMI with significantly lower daily step counts among individuals with obesity compared to those with normal BMIs. However, these authors similarly included one category of obesity, defined as BMI ≥ 30 kg/m^2^ and their median BMI in the obese group was 32.9 kg/m^2^.

One of the few studies to stratify individuals with obesity into specific BMI categories was a cross‐sectional analysis conducted in Sweden, involving 3868 pregnant individuals, aimed at evaluating adherence to the recommended physical activity guideline of at least 150 min per week during pregnancy [26]. Consistent with the methodology of the present study, participants were categorized based on whether or not they met this activity threshold. They reported that approximately 50% of individuals with a normal BMI met the guideline, whereas adherence decreased as BMI increased. Specifically, only 39.2% of those with a BMI ≥ 40 kg/m^2^ met the recommended activity level. It is important to note that their reported activity levels were much higher than those in previous studies. The authors attribute this to having only a single measurement point for activity (10 weeks gestation) during which individuals were also asked to consider their activity over the previous 12 months in their answer. Additionally, the cohort included only 28 individuals classified as having class 3 obesity, limiting the robustness of findings for this subgroup.

Importantly, despite the well‐established benefits of physical activity during pregnancy, participation rates remain suboptimal. Although the lowest levels of activity are seen in individuals with obesity, high levels of physical inactivity are consistently observed across all BMI categories. The underlying factors contributing to these patterns are not yet fully understood and continue to be an area of active investigation. One possible factor contributing to lower activity levels among pregnant individuals with obesity is that, as recently as 2015, ACOG identified “extreme morbid obesity” as a relative contraindication to physical activity [10]. However, the most recent ACOG guidelines no longer include this as a contraindication [27]. Instead, current recommendations emphasize personalization of physical activity rather than restriction, encouraging pregnant individuals with obesity to be physically active.

Strengths of this study include the large sample size, both in the overall cohort and specifically among individuals with class 3 obesity. To our knowledge, this represents the largest study to date evaluating physical activity in pregnant patients with class 3 obesity. Additionally, our cohort encompasses multiple clinical practice settings in the United States. This is important since much of the existing literature has primarily focused on European populations, which appear to be more physically active in general.

This study has several limitations. First, physical activity was self‐reported, which is subject to error, and we were unable to validate their actual activity levels. Participants may have reported what they thought was expected rather than their actual behavior, leading to either under‐ or over‐reporting of activity. Additionally, the study relied on data from a pre‐existing survey that could not be modified. As a result, terms such as “moderate” or “strenuous” were not able to be defined, leaving room for varying interpretation by the respondents. Finally, the survey captured physical activity at a single point in time and did not provide a longitudinal assessment of physical activity across trimesters. However, because the questionnaire data were collected in the first half of the pregnancy, reported activity levels may actually be overestimated since data consistently show that physical activity levels decrease as pregnancy progresses [23].

## 5 CONCLUSION

In conclusion, this study offers valuable insights into physical activity during pregnancy, with a particular focus on individuals with obesity. By stratifying the obese population into class 1, class 2, and class 3 categories—and further stratifying class 3 obesity into more specific BMI subgroups—this study contributes novel data to a largely understudied area. Given the increasing prevalence of class 3 obesity, it is becoming increasingly important to better understand the unique prenatal care needs of this population. The low levels of physical activity observed in all pregnant individuals, particularly those with obesity, may contribute to existing disparities in pregnancy outcomes. Future research should focus on developing and evaluating interventions that promote physical activity during pregnancy, as well as identifying barriers to participation. Such efforts have the potential to improve health outcomes for both the mother and the neonate.

## CONFLICT OF INTEREST STATEMENT

The authors declare no conflicts of interest.

## ETHICS STATEMENT

The IRB determined that the protocol is considered exempt on 7/21/2023 (IRB #22‐008070).

## Data Availability

Data supporting the findings of this study are available from the corresponding author, EN, upon reasonable request.
